# Changes in malaria patterns in Brazil over 28 years (1990–2017): results from the Global Burden of Disease Study 2017

**DOI:** 10.1186/s12963-020-00211-6

**Published:** 2020-09-30

**Authors:** Juliana Maria Trindade Bezerra, David Soeiro Barbosa, Francisco Rogerlândio Martins-Melo, Guilherme Loureiro Werneck, Érika Martins Braga, Pedro Luiz Tauil, Mariângela Carneiro

**Affiliations:** 1 Laboratory of Epidemiology of Infectious and Parasitic Diseases, Department of Parasitology, Institute of Biological Sciences, Universidade Federal de Minas Gerais, Belo Horizonte, Avenida Presidente Antônio Carlos, 6627, Pampulha, Belo Horizonte, Minas Gerais 31270-901 Brazil; 2 Federal Institute of Education, Science, and Technology of Ceará, Rua Francisco da Rocha Martins, S/N, Pabussu, Caucaia, Ceará 61609-090 Brazil; 3 Department of Epidemiology, Social Medicine Institute, Universidade do Estado do Rio de Janeiro, Rua São Francisco Xavier 524, Maracanã, Rio de Janeiro, 20550-013 Brazil; 4 Institute for Public Health Studies, Universidade Federal do Rio de Janeiro, Avenida Horácio Macedo, S/N, Ilha do Fundão – Cidade Universitária, Rio de Janeiro, 21941-598 Brazil; 5 Laboratory of Malaria, Department of Parasitology, Institute of Biological Sciences, Universidade Federal de Minas Gerais, Belo Horizonte, Avenida Presidente Antônio Carlos, 6627, Pampulha, Belo Horizonte, Minas Gerais 31270-901 Brazil; 6 School of Medicine, Postgraduate Program in Tropical Medicine, Universidade de Brasília, Campus Universitário Darcy Ribeiro, Asa Norte, Brasília, Distrito Federal 70910-900 Brazil; 7 Post-Graduation Program in Health Sciences, Infectology and Tropical Medicine, Universidade Federal de Minas Gerais, Avenida Professor Alfredo Balena, 190, Santa Efigênia, Belo Horizonte, Minas Gerais 30130-100 Brazil

**Keywords:** Malaria, Burden of disease, Disability-adjusted life year, Brazil

## Abstract

**Background:**

This study presents the malaria burden in Brazil from 1990 to 2017 using data from the Global Burden of Diseases, Injuries, and Risk Factors Study 2017 (GBD 2017), by analyzing disease burden indicators in federated units of the Legal Amazon and Extra-Amazon regions, as well as describing malaria cases according to *Plasmodium* species occurring in the country.

**Methods:**

We used estimates from the GBD 2017 to report years of life lost due to premature death (YLLs), years lived with disability (YLDs), and disability-adjusted life years (DALYs) for malaria in Brazil, grouped by gender, age group, and Brazilian federated unit, from 1990 to 2017. Results are presented as absolute numbers and age-standardized rates (per 100,000 inhabitants) with 95% uncertainty intervals (UI).

**Results:**

At the national level, the age-standardized DALYs rate due to malaria decreased by 92.0%, from 42.5 DALYs per 100,000 inhabitants (95% UI 16.6–56.9) in 1990 to 3.4 DALYs per 100,000 inhabitants (95% UI 2.7–4.7) in 2017. The YLLs were the main component of the total DALYs rate for malaria in 1990 (67.3%), and the YLDs were the main component of the metric in 2017 (61.8%). In 2017, the highest sex–age DALYs rate was found among females in the “< 1-year-old” age group, with a 6.4 DALYs per 100,000 inhabitants (95% UI 1.8–14.7) and among males in the age group of “20 to 24 years old”, with a 4.7 DALYs per 100,000 inhabitants (95% UI 3.3–9.9). Within the Brazilian Amazon region, the three federated units with the highest age-standardized DALYs rates in 2017 were Acre [28.4 (95% UI 14.2–39.1)], Roraima [28.3 (95% UI 13.5–40.2)], and Rondônia [24.7 (95% UI 11.4–34.8)]. Concerning the parasite species that caused malaria, 73.5% of the total of cases registered in the period had *Plasmodium vivax* as the etiological agent.

**Conclusions:**

The results of the GBD 2017 show that despite the considerable reduction in the DALYs rates between 1990 and 2017, malaria remains a relevant and preventable disease, which in recent years has generated more years of life lost due to disability than deaths. The states endemic for malaria in the Amazon region require constant evaluation of preventive and control measures. The present study will contribute to the direction of current health policies aimed at reducing the burden of malaria in Brazil, as knowing the geographical and temporal distribution of the risk of death and disability of this disease can facilitate the planning, implementation, and improvement of control strategies aimed at eliminating the disease.

## Background

Malaria is an infectious disease with episodic acute manifestations and is caused by protozoan parasites of the genus *Plasmodium* [1, 2]. Five species of *Plasmodium* can infect humans; however, *P. vivax* and *P. falciparum* are the main responsible for causing the disease, with the latter showing a high mortality rate and being predominant in African countries [1–4]. Despite being considered treatable and presenting a favorable evolution when effective health actions are adopted, malaria remains a relevant disease from a global health perspective [5–7].

In 2017, the World Health Organization (WHO) estimated that about 219 million [95% confidence interval (CI) 203–262 million] new cases of malaria had occurred worldwide, corresponding to an increase of two million notifications in relation to the previous year (2016: 217 million, 95% CI 200–259 million) [8].

Regarding the malaria in the Americas, a total of 568,283 confirmed cases were reported in 2016, an increase of 116,000 in relation to the previous year. Between 2010 and 2016, 19 of the 21 endemic countries in the region reported autochthonous transmission of malaria. Brazil, Colombia, Guyana, Haiti, Peru, and Venezuela are responsible for the greatest disease burden, representing 94% of all the cases in the Americas [9, 10].

In Brazil, in the early 1940s, two thirds of the 40 million inhabitants lived in areas endemic for malaria, and between 6 and 8 million infections and around 80,000 malaria-related deaths occurred each year [11–15]. In this context, the National Malaria Service was established, and anti-malaria campaigns were initiated throughout the country [11, 12].

Later, the number of malaria cases increased considerably in the Amazon region as a result of large development projects, such as the construction of roads that facilitated the implementation of ores and timber extraction, livestock, and agricultural settlements, as well as the expansion of colonization and migration of workers to the region [15–17]. Nowadays, malaria still represents a major public health problem in Brazil, with the highest number of cases being registered in the Brazilian Amazon region. In this area, 174,522 cases of malaria have been reported from January to November 2017, which represents an increase of 48% in relation to the same period in 2016, when 117,832 cases of the disease were reported. These have been caused mainly by *P. vivax*, followed by *P. falciparum*, and, to a smaller extent, *P. malariae* [18].

The Global Burden of Disease (GBD) is a descriptive epidemiological study that, since the 1990s, has been quantifying and comparing the magnitude of health losses due to diseases and injuries and identifying risk factors associated with location, sex, age, and year [19, 20]. The GBD study uses the disability-adjusted life year (DALYs), a measure of the health loss due to fatal and nonfatal disease burden, as the population’s primary health metric. The DALYs are estimated by the sum of years lived with disabilities (YLDs) and years of life lost (YLLs) due to premature death for a given cause [19–25].

To the best of our knowledge, this is the first national analysis of the malaria burden stratified by sex, age group, and federated units in Brazil, one of the American countries most affected by the disease. Although the burden of malaria in Brazil has been quantified at the national level, there are clear disparities among different regions, and subnational analyses are needed. Considering that most reports of malaria in Brazil result from *P. vivax* infection, over the years, it is observed that, although the disease does not have a high mortality rate, it affects a significant number of cases with great social and economic impact. The analysis of the burden of malaria goes beyond the knowledge about the incidence and mortality of the disease and can be considered an additional tool for understanding this health problem. Therefore, the results of the GDB will allow a better understanding of the country’s malaria burden, a key step in policy implementation as it leads to the compression of morbidity caused by the disease. Identifying levels and trends of malaria burden can assist health authorities in planning interventions, monitoring processes, and assessing the impact and effectiveness of adopted disease control measures. In the present study, we analyzed the malaria burden in Brazil and its 27 federated units using the data from the GBD 2017 study describing the main disease burden metrics DALYs, YLDs, and YLLs, in federated units in the Legal Amazon and Extra-Amazon regions, as well as describing malaria cases according to *Plasmodium* species occurring in the country.

## Methods

### Study area

The Federative Republic of Brazil is the largest country in South America and the fifth largest worldwide in territorial area, with 8.5 million Km^2^ (equivalent to 47% of the South American territory) [26]. Regarding population size, the country is the second largest in the Americas and the sixth in the world, with approximately 210 million inhabitants in 2018 [26].

In this study, we present results for malaria burden at the national level and for all the 27 federated units. The data are presented considering the five geographic regions (North, Northeast, Central-West, Southeast, and South) [27], as well as the Legal Amazon (Acre—AC, Amapá—AP, Amazonas—AM, Pará—PA, Rondônia—RO, Roraima—RR, Tocantins—TO, Maranhão—MA, and Mato Grosso—MT) and the non-endemic Extra-Amazon (Alagoas—AL, Bahia—BA, Ceará—CE, Paraíba—PB, Pernambuco—PE, Piauí—PI, Rio Grande do Norte—RN, Sergipe—SE, Distrito Federal—DF, Goiás—GO, Mato Grosso do Sul—MS, Espírito Santo—ES, Minas Gerais—MG, Rio de Janeiro—RJ, São Paulo—SP, Paraná—PR, Rio Grande do Sul—RS, and Santa Catarina—SC) [27, 28].

We compared the malaria estimates from the GBD 2017 study for 2017 with those generated in 1990. These estimates are available on the GBD study platform at http://vizhub.healthdata.org/gbd-compare and http://ghdx.healthdata.org/gbd-results-tool.

### GBD overview

The GBD study is coordinated by the Institute for Health Metrics and Evaluation at the University of Washington, USA [24] and consist of a systematic and scientific effort to quantify the comparative magnitude of health losses due to diseases, injuries, and risk factors by sex, age, and location over time [25]. The general methodological approaches used to estimate the metrics in the GBD 2017 are detailed in previous publications [19–23]. The GBD study uses the disability-adjusted life years (DALYs) of a given population as the main health metric. The DALYs results from the sum of the years of disability (YLDs) and the years of life lost due to premature death (YLLs) [22]. A DALY represents 1 year of healthy life lost due to a specific illness or injury [22].

Herein, we used the data and estimates from the GBD 2017 to explore the malaria burden in Brazil from 1990 to 2017. The GBD 2017 provides a comprehensive annual assessment of mortality and morbidity estimates for more than 300 diseases and injuries and 84 risk factors for 195 countries and territories from 1990 to 2017 [21, 22, 24, 25]. The GBD 2017 list of hierarchy of disease causes is organized into four levels, which are mutually exclusive and collectively exhaustive [24, 25]. The cause of malaria was defined and identified according to the International Classification of Diseases, 9th (ICD-9) and 10th (ICD-10) Revisions: ICD-9 (Code 084), ICD-9 BTL (Code B052), and ICD-10 (Codes B50-B54, P37.3, P37.4) [29].

The GBD data sources for Brazil have been described elsewhere [30–34]. The mortality data and the data used to generate the YLLs estimates came from the Brazilian Mortality Information System (*Sistema de Informações sobre Mortalidade—SIM*) and were adjusted by other national and international sources. The main sources of morbidity data, used for the estimation of the YLDs, were the Information System of Diseases Notification (*Sistema de Informação de Agravos de Notificação—SINAN*), the Epidemiological Surveillance Information System—Case Report (*Sistema de Informação de Vigilância Epidemiológica* - *Notificação de casos*—SIVEP/Malária), the Hospital Information System of the Unified Health System (*Sistema de Informações Hospitalares do Sistema Único de Saúde—SIH/SUS*), and the Outpatient Information System of the Unified Health System (*Sistema de Informações Ambulatoriais do Sistema Único de Saúde—SIA/SUS*) [30–32]. Additional data on the prevalence of diseases from published Brazilian population-based studies and databases from control programs were also used in the GBD study [30–32].

In the GBD study, each death is attributed to a single underlying cause, which is the cause that initiated the series of events that led to death, in accordance with the ICD principles [20]. In the GBD 2017, mortality sub-registration and the redistribution of “garbage codes” were corrected based on the GBD 2017 redistribution algorithms [20]. “Garbage codes” are the assignment of causes of death that could not or should not be classified as the underlying cause of death [20]. The GBD 2017 used the Cause of Death Ensemble model (CODEm), negative binomial regression, and natural history models to estimate the number of causes of deaths for neglected tropical diseases (NTDs) by location, sex, age, and year. The modeling strategy that they used for morbidity estimation and validation has been published elsewhere [22]. All available data that the GBD study uses is required to meet a minimum standard of acceptable quality for each disease. The GBD 2017 used the Bayesian regression analytic tool DisMod-MR 2.1 to synthesize consistent estimates of prevalence and incidence of nonfatal outcomes by sex, age, year, and location using a wide range of updated and standardized analytical procedures [22].

The burden of malaria was assessed by the metrics of incidence, number of deaths, years of life lost due to premature death (YLLs), years lived with disability (YLDs), and disability-adjusted life year (DALYs = YLLs + YLDs). The YLLs express the effect of premature deaths on the population and results from the multiplication of the number of deaths due to malaria at a certain age by the standard life expectancy. The GBD 2017 considered a global standard life expectancy of 72.9 (95% UI 72.6–73.2) years at birth, based on the lowest observed death rates for each 5-year age range in 2017 [35]. The YLDs express the sum of the prevalence of sequelae related to malaria multiplied by the disability weight [22]. The disability weight reflects the severity of health loss associated with a given disease and is presented on a scale varying from 0 (perfect health) to 1 (equivalent to death) [22]. The sum of the YLLs and the YLDs yields the DALYs [22].

Herein, we present the estimates as age-standardized rates by 100,000 inhabitants. The age-standardized rates were calculated using the GBD’s world population standard. The metrics are presented with their respective 95% uncertainty intervals (95% UI) and the relative percentages of change. We also present the annual rates of change (ARC) estimated by the GBD 2017, to highlight the variations between 1990 and 2017 [36].

We ranked the federated units from the highest to the lowest value of DALYs for malaria. Additionally, we used the software QGIS version 2.18.2 (Las Palmas, Spain) to create thematic maps, which were used to show the spatial distribution of the metrics DALYs, YLLs, and YLDs, and the percentages of change over time.

### Brazilian malaria data reports and species of infecting parasites

Since the GBD 2017 does not estimate the malaria burden for each species of *Plasmodium*, we assessed the number of confirmed cases of the disease considering the species of the etiological agent over the years. In our analyses, we considered the infections caused by *P. vivax*, *P. falciparum*, and “others” (which include co-infection with *P. vivax* and *P. falciparum*, infections by *P. malariae*, infections by *P. ovale*, and infections by non *P. falciparum* species, according to the Brazilian Ministry of Health classification data).

The graph of cases by species over time was plotted using three main sources of information: The number of disease cases confirmed in the country between 1990 and 2002 were obtained from (i) the SUS Epidemiological Report (*Informe Epidemiológico do SUS*) [37], available at http://scielo.iec.gov.br/scielo.php?script=sci_arttext&pid=S0104-16731997000100004 and (ii) the National Health Foundation (*Fundação Nacional de Saúde—FUNASA*) [38], available at http://www.funasa.gov.br/epi/malaria/malaria0.htm, while the notifications from 2003 to 2017 were collected from (iii) the Epidemiological Surveillance Information System—Case Report (*Sistema de Informação de Vigilância Epidemiológica* - *Notificação de casos*—SIVEP/Malária) [39], available at http://200.214.130.44/sivep_malaria/.

### Ethical considerations

The protocol for this study was approved by the Research Ethics Committee of the Federal University of Minas Gerais, Belo Horizonte, Brazil (Project CAAE 62803316.7.0000.5149).

## Results

### GBD estimates at the national level

The main metrics on malaria burden and relative change from 1990 to 2017 in Brazil are presented in Table 1. In 2017, the incidence rate was 97.2 cases per 100,000 inhabitants (95% UI 80.4–117.2), and the mortality rate was 0.02 deaths per 100,000 inhabitants (95% UI 0.01–0.06), representing a reduction of 96.4% and 95.0%, respectively, in comparison with 1990. The DALYs rate also decreased in the country, going from 42.5 DALYs per 100,000 inhabitants (95% UI 16.6–56.9) in 1990 to 3.4 DALYs per 100,000 inhabitants (95% UI 2.7–4.7) in 2017, representing a percentage change of − 92.0% and an ARC of − 9.3% (Table 1).

**Table 1:** Incidence rates, number of deaths, years of life lost due to premature death (YLLs), years lived with disability (YLDs), and disability-adjusted life years (DALYs) for malaria in Brazil in 1990 and 2017

**Metrics**	**Absolute number** ***N*** **(95% UI)**			**Rate per 100,000 inhabitants (95% UI)**			
	**1990**	**2017**	**Relative change (%)**	**1990**	**2017**	**Relative change (%)**	**ARC 1990–2017 (%)**
Incidence	4612,232 (2,315,926–8,359,156)	209,669 (173,992–251,852)	− 95.4	2700.1 (1387.8–4827.4)	97.2 (80.4–117.2)	− 96.4	− 12.3
Deaths	670 (123–852)	58 (42–145)	− 91.3	0.4 (0.08–0.5)	0.02 (0.01–0.06)	− 95.0	− 10.2
YLLs	47,423 (8249–61,500)	2802 (2101–6282)	− 94.0	28.6 (4.9-36.9)	1.3 (1.0–2.9)	− 95.4	− 11.3
YLDs	22,968 (11,768–41,202)	4323 (3265–5552)	− 81.1	13.9 (7.2–24.6)	2.1 (1.5–2.6)	− 85.6	− 7.0
DALYs	70,391 (27,531–94,759)	7125 (5672–10,218)	− 89.8	42.5 (16.6–56.9)	3.4 (2.7–4.7)	− 92.0	− 9.3

Absolute numbers and age-standardized rates per 100,000 inhabitants are presented, along with relative change and annual rate of change (ARC), 1990–2017.

*N* absolute value of the metric, *95% UI* uncertainty interval of 95%, % percentage, *YLLs* years of life lost due to premature death, *YLDs* years lived with disability, *DALYs* disability-adjusted life years, *ARC* annual rate of change

**Fig. 1:**
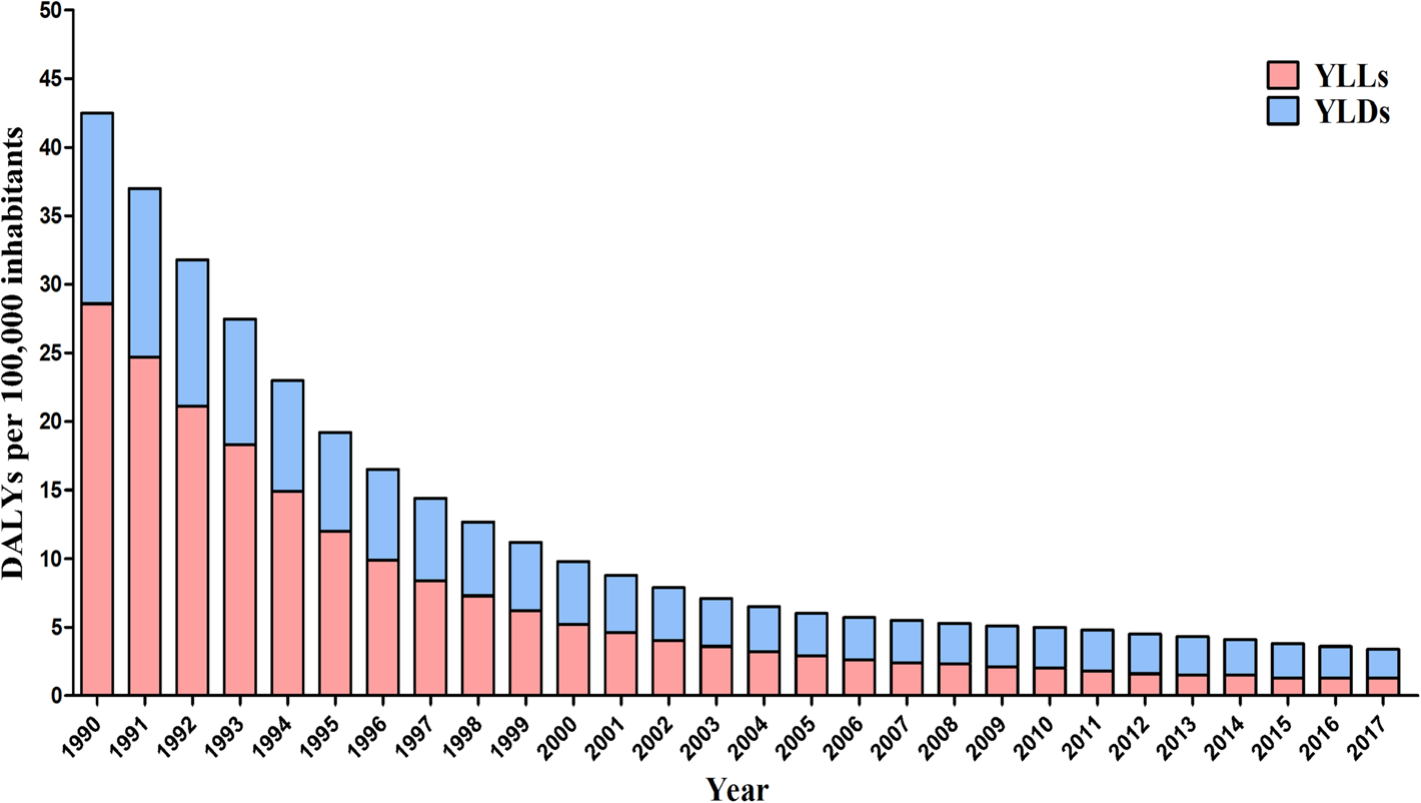
Disability-adjusted life years (DALYs) for malaria. Age-standardized rates per 100,000 inhabitants are presented, along with the contribution of the rates of years of life lost due to premature death (YLLs) and years lived with disability (YLDs). Brazil, 1990–2017

In 1990, the YLLs rate [28.6 (95% UI 4.9–36.9) per 100,000 inhabitants] accounted for 67.3% of the DALYs, while the YLDs rate [13.9 (95% UI 7.2–24.6) per 100,000 inhabitants] corresponded to 32.7% of the total DALYs due to malaria. Over the years, the contributions of the YLLs and the YLDs to the total DALYs of malaria changed. In 2017, 38.2% of the DALYs rate corresponded to the YLLs [1.3 (95% UI 1.0–2.9) per 100,000 inhabitants], and 61.8% corresponded to the YLDs [2.1 (95% UI 1.5–2.6) per 100,000 inhabitants] (Fig. 1).

### GBD estimates by sex and age groups

**Fig. 2:**
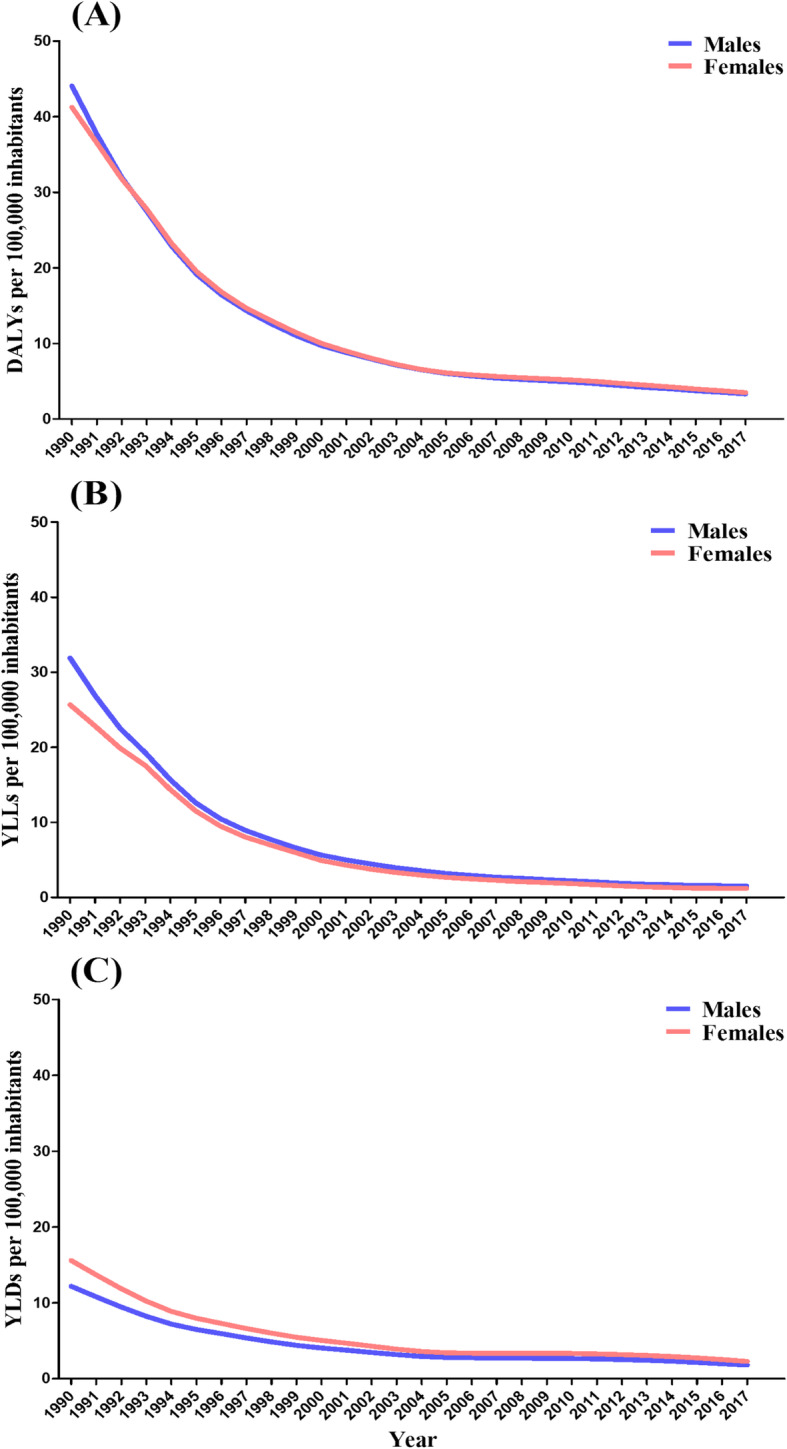
Age-standardized rates per 100,000 inhabitants of disability-adjusted life years (DALYs) (**a**), years of life lost due to premature death (YLLs) (**b**), and years lived with disability (YLDs) (**c**) for malaria, according to sex. Brazil, 1990–2017

Over the years, the DALYs, YLLs, and YLDs rates were similar between genders. In the early 1990s, males presented higher values of DALYs and YLLs (Fig. 2a and b) in contrast with females, who presented higher values of YLDs in the same period (Fig. 2c). A marked decrease was observed in the three metrics between 1990 and 2017. The DALYs rates for males and females were less than 10 per 100,000 inhabitants from the year 2000 onwards (Fig. 2a). Regarding the YLLs and YLDs, rates lower than 10 per 100,000 inhabitants were verified in 1996 and 1992, respectively (Fig. 2b and c).

**Fig. 3:**
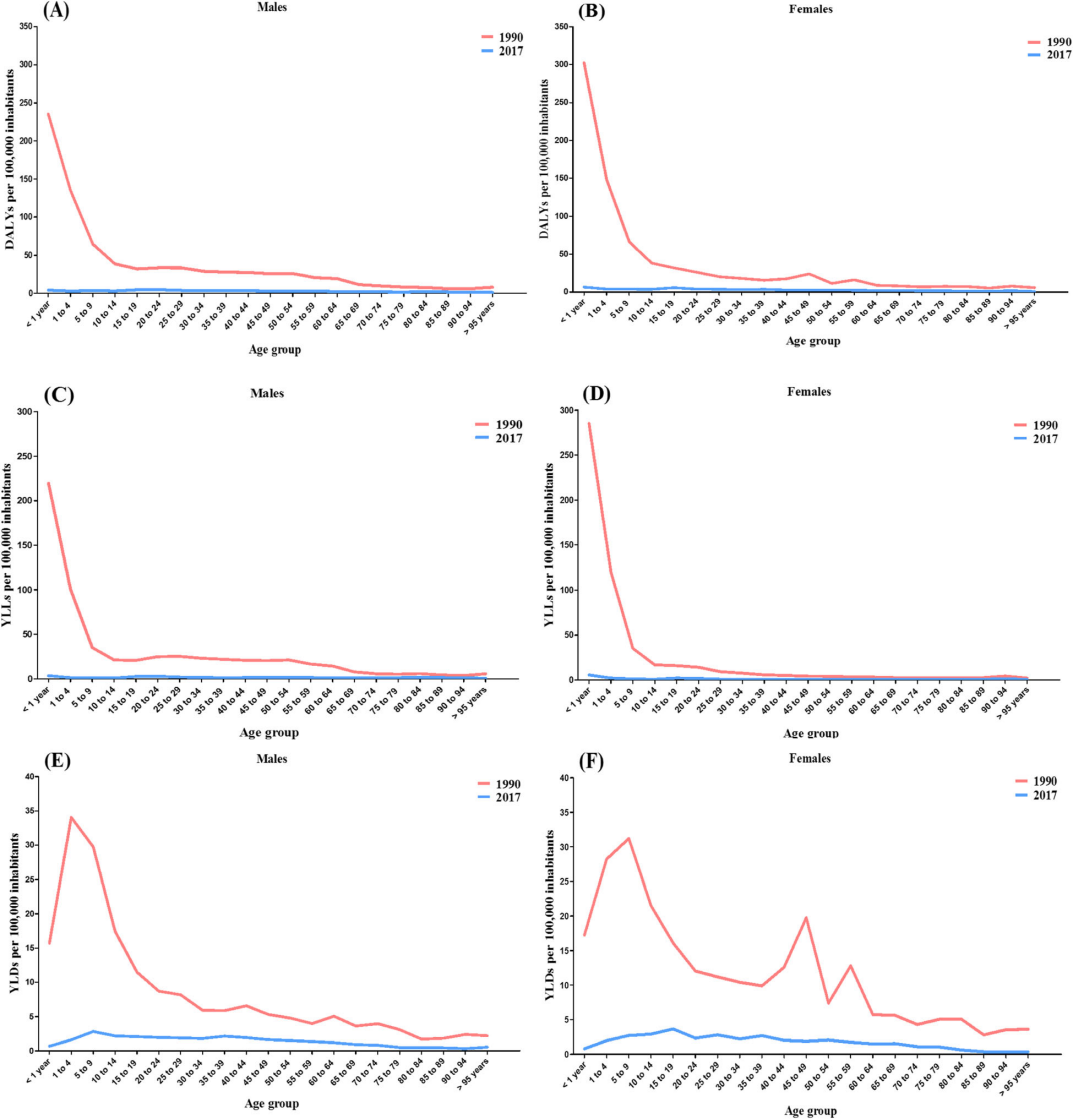
Age-standardized rates per 100,000 inhabitants of disability-adjusted life years (DALYs), years of life lost due to premature death (YLLs), and years lived with disability (YLDs), according to sex and age group. Brazil, 1990–2017. DALYs per 100,000 inhabitants for males (**a**) and females (**b**) at each age group. YLLs per 100,000 inhabitants for males (**c**) and females (**d**) at each age group. YLDs per 100,000 inhabitants for males (**e**) and females (**f**) at each age group

The year 1990 also showed the highest rates per 100,000 inhabitants for the DALYs, YLLs, and YLDs, for both males and females, in all age groups. In that year, the highest DALYs rate was found in the age group of “< 1 year old” for both sexes, being 235.3 (95% UI 24.9–360.9) for males (Fig. 3a) and 302.3 (95 % UI 20.7–512.3) for females (Fig. 3b). This age group (“< 1 year old”) also showed the highest YLLs rates: 219.6 (95% UI 11.7–345.1) for males (Fig. 3c) and 285.0 (95% UI 6.2–494.7) for females (Fig. 3d). The indicator YLLs presented the highest rates among males in the age group of “1 to 4 years old” [34.0 (95% UI 12.2–76.3)] (Fig. 3e) and among females in the age group of “5 to 9 years old” [31.2 (95% UI 10.8–74.3)] (Fig. 3f).

In 2017, the highest DALYs rates were observed among males in the age group of “20 to 24 years old” [4.7 (95% UI 3.3–9.9)] (Fig. 3a) and among females “< 1 year old” [6.4 (95% UI 1.8–14.7)] (Fig. 3b). The highest YLLs rates for both sexes were found in the age group of “< 1 year old”, being 3.4 (95% UI 1.5–10.5) for males (Fig. 3c) and 5.6 (95% UI 1.1–13.6) for females (Fig. 3d). Regarding the YLDs, the age group with the highest rate among males was that of “5 to 9 years old” [(2.8 (95% UI 1.6–4.9)] (Fig. 3e) and that of “15 to 19 years old” [3.6 (95% UI 2.2–6.2)] among females (Fig. 3f).

### GBD estimates by federated units

From 1990 to 2017, the incidence and DALYs rates per 100,000 inhabitants for malaria reduced in all federated units. The highest incidence and DALYs rates per 100,000 inhabitants were found in most of the states in the Legal Amazon region both in 1990 and 2017. In terms of the incidence rates, the state of Acre (in the North of Brazil and within the Legal Amazon region) presented the highest rates per 100,000 inhabitants both in 1990 [94,748.3 (95% UI 49,564.6–172,108.5)] and in 2017 [3296.0 (95% UI 2677.7–4074.3)]. The highest DALYs rates were detected in the state of Rondônia (also located in the North region and within the Legal Amazon area) in 1990 [1243.1 (95% UI 583.5–1788.2)] and in the state of Acre in 2017 [28.5 (95% UI 14.3–39.1)] (Table 2).

**Table 2:** Incidence and DALYs rates for malaria, according to federated units and regions

	**Incidence per 100,000 inhabitants (95% UI)**			**DALYs per 100,000 inhabitants (95% UI)**		
**Regions**	**1990**	**2017**	**Relative change (%)**	**1990**	**2017**	**Relative change (%)**
Legal Amazon*						
North**						
Acre	94,748.3 (49,564.6–172,108.5)	3296.0 (2677.7–4074.3)	− 96.5	1048.1 (328.5–1546.2)	28.5 (14.3–39.1)	− 97.2
Amapá	31,556.8 (14,412.0–64,852.0)	2335.5 (1894.6–2805.1)	− 92.5	469.7 (70.5–655.5)	24.5 (11.8–34.0)	− 94.7
Amazonas	46,631.1 (24,532.6–83,569.1)	2029.4 (1609.0–2495.3)	− 95.6	257.4 (113.2–443.0)	14.2 (10.1–20.3)	− 94.4
Pará	18,978.0 (10,098.0–33,150.1)	373.1 (305.2–456.6)	− 98.0	401.0 (60.4–568.7)	13.1 (6.6–17.4)	− 96.7
Rondônia	90,883.6 (44,462.1–178,645.5)	1332.5 (1060.3–1610.7)	− 98.5	1243.2 (583.5–1788.2)	24.7 (11.4–34.9)	− 98.0
Roraima	90,107.4 (45,008.0–163,702.4)	2717.6 (2185.9–3350.8)	− 96.9	1080.9 (285.1–1591.3)	28.3 (13.6–40.3)	− 97.3
Tocantins	171.1 (83.3–318.2)	3.7 (3.0–4.6)	− 97.8	46.1 (6.1–80.8)	5.6 (2.9–7.3)	− 87.8
Northeast**						
Maranhão	2306.8 (1120.2–4306.2)	34.5 (28.6–41.6)	− 98.5	27.1 (13.8–47.3)	4.4 (3.3–5.7)	− 83.7
Central-West**						
Mato Grosso	2351.8 (1139.6–4243.4)	48.5 (39.0–61.4)	− 98.1	250.7 (13.2–360.8)	8.2 (3.4–11.2)	− 96.7
Extra-Amazon*						
Northeast**						
Alagoas	66.9 (23.0–161.1)	0.1 (0.09–0.2)	− 99.8	4.3 (2.8–9.0)	1.9 (1.2–4.6)	− 55.8
Bahia	33.9 (13.3–74.8)	0.1 (0.09–0.2)	− 99.7	4.2 (3.1–7.2)	1.9 (1.2–4.4)	− 54.7
Ceará	10.0 (4.8–18.6)	0.3 (0.2–0.4)	− 97.0	4.2 (2.9–7.1)	1.9 (1.3–4.0)	− 54.7
Paraíba	6.2 (2.9–11.4)	0.2 (0.1–0.3)	− 96.7	3.8 (2.5–6.9)	1.9 (1.2–4.1)	− 50.0
Pernambuco	31.9 (13.3–67.5)	0.1 (0.09–0.2)	− 99.6	4.3 (2.9–8.6)	1.9 (1.2–4.4)	− 55.8
Piauí	163.2 (75.2–322.1)	1.8 (1.5–2.1)	− 98.8	7.0 (5.1–9.5)	2.2 (1.6–3.9)	− 68.5
Rio Grande do Norte	16.8 (8.3–29.7)	0.4 (0.3–0.5)	− 97.6	4.6 (3.4–6.8)	2.0 (1.3–4.4)	− 56.5
Sergipe	36.7 (15.7–75.6)	0.2 (0.1–0.3)	− 99.4	4.4 (3.1–7.9)	2.0 (1.3–4.5)	− 54.5
Central-West**						
Distrito Federal	20.4 (8.6–42.4)	1.0 (0.8–1.2)	− 95.0	9.8 (5.2–12.4)	2.1 (1.4–3.7)	− 78.5
Goiás	32.0 (14.2–65.5)	1.3 (1.0–1.6)	− 95.9	29.1 (5.4–39.3)	2.7 (2.0–4.5)	− 90.7
Mato Grosso do Sul	18.6 (8.4–36.6)	0.8 (0.6–0.9)	− 95.6	11.2 (5.0–14.8)	2.2 (1.6–4.2)	− 80.3
Southeast**						
Espírito Santo	39.7 (17.7–81.9)	1.6 (1.3–1.9)	− 95.9	6.1 (4.6–8.6)	2.2 (1.5–4.5)	− 63.9
Minas Gerais	21.8 (10.2–40.9)	0.4 (0.3–0.5)	− 98.1	4.3 (3.1–7.0)	1.9 (1.2–4.1)	− 55.8
Rio de Janeiro	9.6 (4.7–17.6)	0.5 (0.4–0.6)	− 94.7	4.3 (3.0–8.4)	1.9 (1.2–4.4)	− 55.8
São Paulo	12.4 (6.1–22.4)	0.5 (0.4–0.6)	− 95.9	4.4 (3.2–7.2)	1.8 (1.2–3.7)	− 59.0
South**						
Paraná	14.3 (6.5–29.7)	0.6 (0.5–0.8)	− 95.8	7.1 (4.8–9.0)	2.0 (1.4–4.2)	− 71.8
Rio Grande do Sul	4.0 (1.7–80.7)	0.1 (0.09–0.2)	− 97.5	4.0 (2.9–6.5)	1.9 (1.2–4.1)	− 52.5
Santa Catarina	8.7 (3.8–17.9)	0.3 (0.2–0.4)	− 96.5	5.4 (4.1–7.4)	2.0 (1.3–3.9)	− 62.9

Age-standardized rates per 100,000 inhabitants are shown, along with the relative changes. Brazil, 1990–2017

*95% UI* uncertainty interval of 95%, *%* percentage

* Malaria transmission areas in Brazil, considering the Legal Amazon region, comprising nine states in the Brazilian geographic regions North, Northeast, and Central-West (Acre, Amapá, Amazonas, Pará, Rondônia, Roraima, Tocantins, Maranhão, and Mato Grosso), and the Extra-Amazon region, comprising the other 17 states and the Federal District located in the geographic regions Northeast, Central-West, Southeast, and South (Alagoas, Bahia, Ceará, Paraíba, Pernambuco, Piauí, Rio Grande do Norte, Sergipe, Distrito Federal, Goiás, Mato Grosso do Sul, Espírito Santo, Minas Gerais, Rio de Janeiro, São Paulo, Paraná, Rio Grande do Sul, and Santa Catarina)

**The five Brazilian geographic regions: North, Northeast, Central-West, Southeast, and South

Among the states located in the Extra-Amazon region, Piauí (in the Northeast of Brazil) stood out as presenting the highest incidence rates per 100,000 inhabitants in 1990 [163.2 (95% UI 75.2–322.1)] and in 2017 [1.8 (95% UI 1.5–2.1)], showing a percentage of change of − 98.8%. The highest DALYs rates were verified in the state of Goiás (Central-West Brazil) in 1990 [29.1 (95% UI 5.4–39.3)] and in 2017 [2.7 (95% UI 2.0–4.5)], showing a percentage change of − 90.7% (Table 2).

**Fig. 4:**
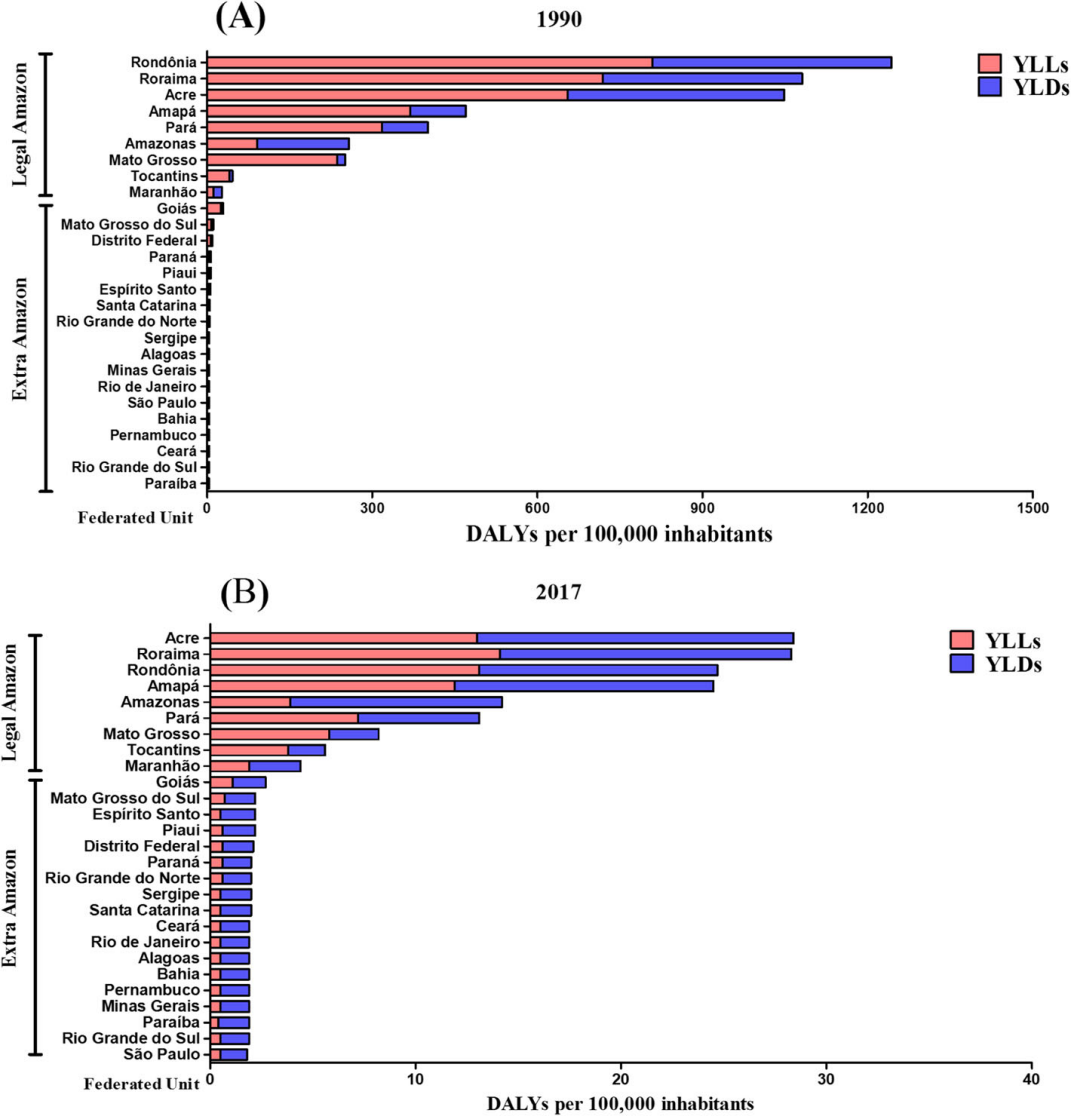
Age-standardized rates per 100,000 inhabitants of disability-adjusted life years (DALYs) due to malaria according to federated unit in the Legal Amazon and the Extra-Amazon regions in the years of 1990 (**a**) and 2017 (**b**). The contributions of the years of life lost due to premature death (YLLs) and the years lived with disability (YLDs) rates are presented. Brazil, 1990–2017

**Fig. 5:**
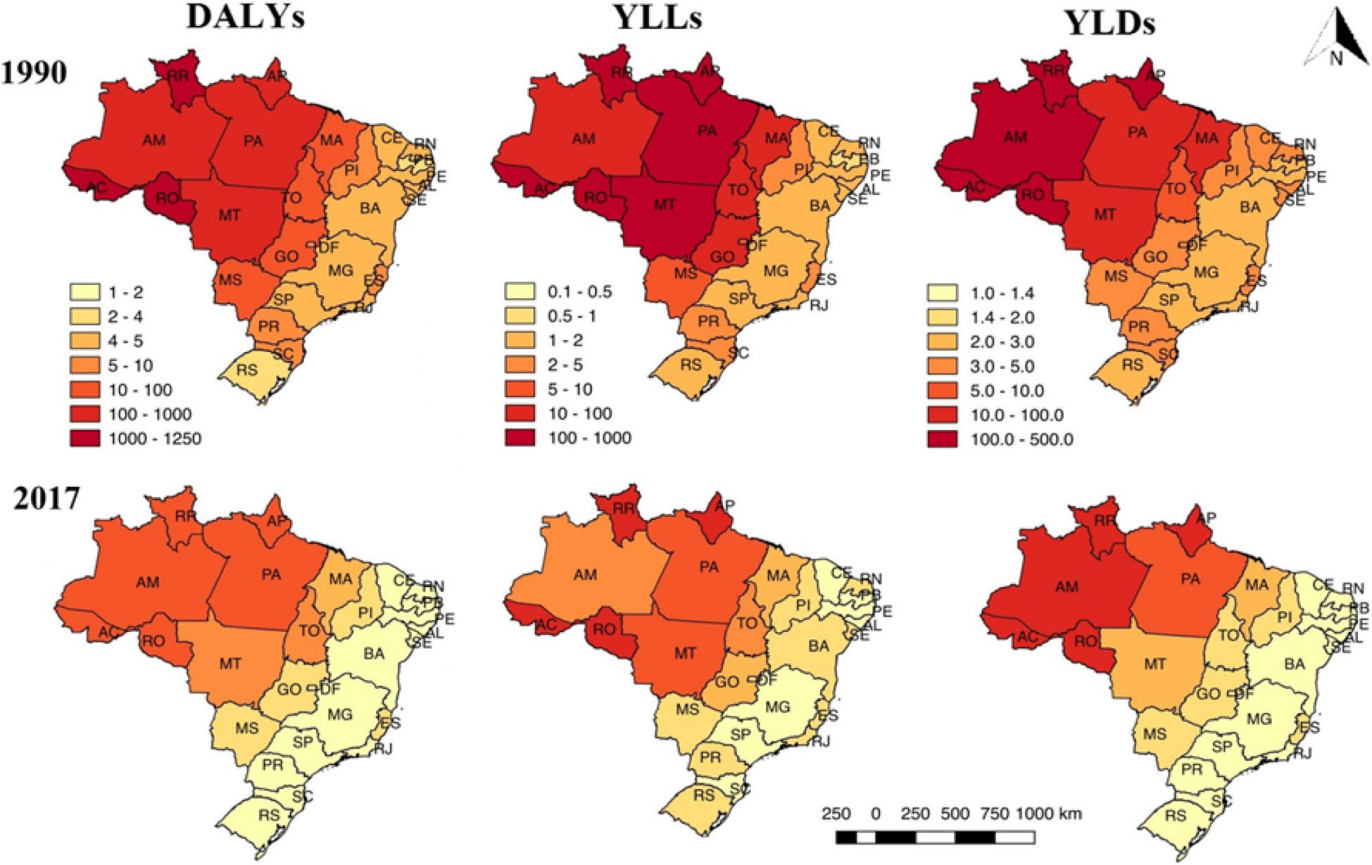
Age-standardized rates per 100,000 inhabitants of disability-adjusted life years (DALYs), years of life lost (YLLs), and years lived with disability (YLDs) due to malaria according to the federated unit. Brazil, 1990–2017

In 1990, the three most expressive DALYs rates ​were found in the states of Rondônia [1243.2 (95% UI 583.5–1788.2)], Roraima [1080.9 (95% UI 285.1–1591.3)], and Acre [1048.1 (95% UI 328.5–1546.2)], all of which are located in the North of Brazil and within the Legal Amazon. In these three states, the YLLs represented the greatest contribution to the DALYs in that year, contributing with 65.0% in Rondônia, 66.4% in Roraima, and 62.4% in Acre (Figs. 4a and 5). In 2017, the same three states showed the highest DALYs rates, albeit ranking at different positions: Acre ranked first [28.5 (95% UI 14.3–39.1)], followed by Roraima [28.3 (95% UI 13.6–40.3)] and Rondônia [24.7 (95% UI 11.4–34.9)]. In the first two, the greatest contribution to the DALYs came from the YLDs, with an observed contribution of 54.2% in the state of Acre and 50.1% in the state of Roraima. In the state of Rondônia, on the other hand, the YLLs contributed with 53.0% of the DALYs (Figs. 4b and 5). In all the federated units, a decrease in the DALYs rates was observed from 1990 to 2017 (Figs. 4a and b and 5).

Among the states located in the Extra-Amazon region, Goiás (in the geographic region, Central-West) presented the highest DALYs rates per 100,000 inhabitants both in 1990 [29.1 (95% UI 5.4–39.3)] and in 2017 [2.7 (95% UI 2.0–4.5)]. The YLLs contributed with 87.2% of the DALYs in 1990, and the YLDs contributed with 61.5% of the DALYs in 2017 (Figs. 4a and b and 5).

**Fig. 6:**
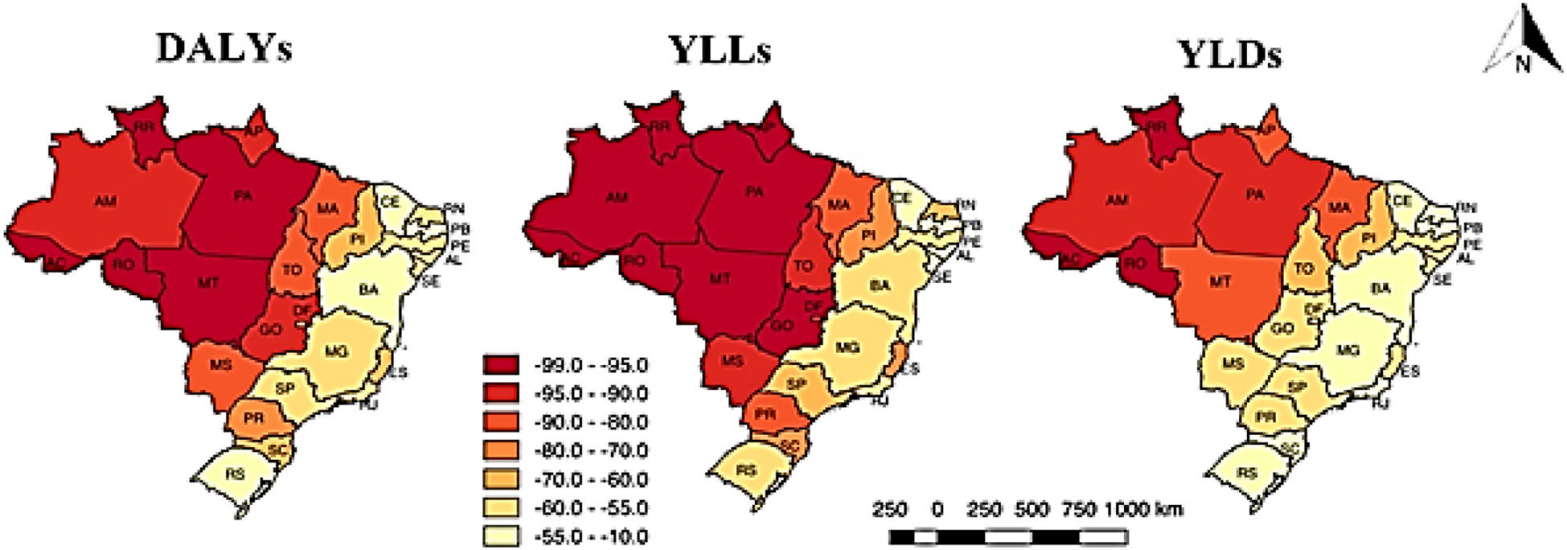
Percentages of change calculated for the disability-adjusted life years (DALYs), years of life lost (YLLs), and years lived with disability (YLDs) rates per 100,000 inhabitants due to malaria according to the federated unit. Brazil, 1990–2017

Among the federated units within the Legal Amazon region, the three with the highest percentage of change over the years were Rondônia, Roraima, and Acre. This observation was consistent for the three metrics: DALYs [(Rondônia (− 98.0%), Roraima (− 97.4%), and Acre (− 97.3%)], YLLs [Rondônia (− 98.4%), Roraima (− 98.0%), and Acre (− 98.0%)], and YLDs [Rondônia (− 97.3%), Roraima (− 96.1%), and Acre (− 96.1%)] (Fig. 6).

In the Extra-Amazon region, the state of Piauí (Northeast Brazil) presented the highest percentage of change for YLDs (− 65.7%), while Goiás (Central-West Brazil) showed the highest percentages of change for YLLs (− 95.5%) and DALYs (− 90.9%) (Fig. 6).

### Brazilian estimates: number of cases of malaria by etiological agent

**Fig. 7:**
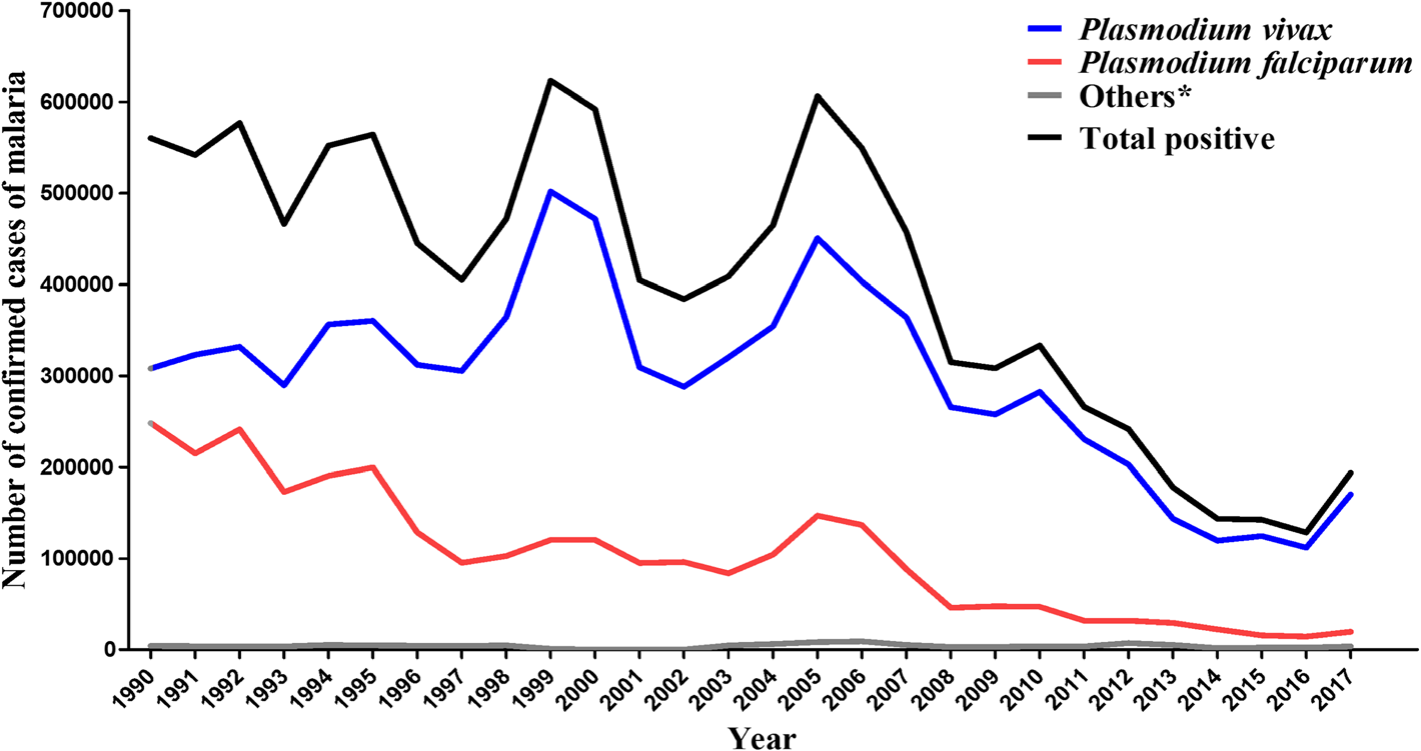
Number of confirmed cases of malaria according to *Plasmodium* species. Brazil, 1990–2017. Sources: Brazilian Ministry of Health [37–39], National Health Foundation (*Fundação Nacional de Saúde*—FUNASA), and Epidemiological Surveillance Information System—Case Report (*Sistema de Informação de Vigilância Epidemiológica—Notificação de casos*—SIVEP/Malária). *Others = co-infections with *P. vivax* and *P. falciparum*, infections caused by *P. malariae* and *P. ovale*, and infections caused by non *P. falciparum*, according to the Brazilian Ministry of Health data classification

Between 1990 and 2017, 11,327,462 cases of malaria were confirmed in Brazil. With regards to the parasite species that caused the disease, 73.5% of the total number of cases had *P. vivax* as the etiological agent, while 25.5% were caused by *P. falciparum*, and 1.0% was caused by other species. The years of 1999 and 2005 had the highest number of cases in the historical series (Fig. 7).

## Discussion

To the best of our knowledge, this is the first comprehensive effort to analyze the burden of malaria in Brazil using data from the GBD 2017, comprising a period of 28 years (from 1990 to 2017) and examining both genders at different age groups and in all the Brazilian federated units (both in the Legal Amazon and the Extra-Amazon regions). The present study showed a reduction in all disease burden indicators in the country during the study period, with a 92.0% decrease in the DALYs per 100,000 inhabitants, a 95.0% decrease in mortality, and a 96.4% decrease in disease incidence. Reductions were evidenced for both sexes, at all age groups, and in all the federated units of the country. An important feature associated with this change was the decrease in the contribution of YLLs to the DALYs rate. In 1990, the YLLs contributed with 67.2% of the DALYs rate, a percentage that decreased to 38.2% in 2017, thus reaffirming the substantial reduction in the mortality due to malaria, which was reflected in the decrease of the YLLs.

Altogether, the DALYs, YLLs, and YLDs gather information on mortality and morbidity and allow the estimation of the impact of each disease or injury on the health status of the population. Therefore, these indicators constitute remarkable tools for policy making aimed at reducing the burden of a disease [19–25].

The fact that Brazil is among the countries with the highest DALYs rates due to malaria in South America [40] highlights a situation that deserves attention. Despite the decrease in all the disease burden indicators between 1990 and 2017 (and particularly in the YLLs rate, which decreased by 95.4% in the period), we noted that the years of life lost due to disability (YLDs) currently constitute the most representative contribution to the DALYs estimates. This scenario may also be a reflex of the decrease in incidence and mortality rates due to the disease observed in the country over the years of study.

We observed that the DALYs, YLLs, and YLDs values over the years were comparable between genders. However, in some geographic regions, men are at a higher occupational risk of contracting malaria as they frequently work in mines, fields, or forests at times of intense activity of the vectors—mosquitoes of the genus *Anopheles* that transmit the parasites to humans [15, 41, 42].

Nevertheless, we observed differences when the age groups were analyzed according to gender. The reasons underlying the high rates of DALYs, YLLs, and YLDs observed among children in 1990 are still poorly understood. However, it should be noted that their immune systems are more susceptible to infections, which is likely one of the reasons why children are more affected than other age groups [43]. On the other hand, the higher DALYs rate observed in 2017 among males in the age group of “20 to 24 years old” may be related to the occupational exposure or to the migration of individuals from this age group to high-risk areas [43, 44].

Noteworthy, the large contribution of the YLDs to the DALYs rate due to malaria in recent years in Brazil reflects the fact that this disease incapacitates those affected to carry out their routine activities (work and school, among others). Studies have shown that most cases of malaria in Brazil affect the economically productive age groups (individuals between 15 and 45 years old), thus indicating that the disease has an economic impact on the affected families due to the loss of productive hours of the patient. Therefore, greater investments are needed to control malaria, not only to reduce its impacts on individuals’ health but also to minimize the economic pressure that it exerts on the population [45–47].

Among the Brazilian federated units, Acre, Rondônia, and Roraima, situated in the Amazon region (in the North of Brazil) had the highest incidence and DALYs rates in 1990 and 2017. The Brazilian Amazon, so-called Legal Amazon (*Amazônia Legal*) since 1953, is formed by the states of Acre, Amapá, Amazonas, Pará, Rondônia, Roraima, Tocantins, and part of the states of Maranhão and Mato Grosso). It presents environments that are favorable to malaria transmission, with risk factors that include poor demographic and socioeconomic conditions, and areas of expansion of the agricultural frontier, logging, road construction, and hydroelectric plants [11, 48–51].

The estimates of the GBD 2017 showed that in the 1990s the highest DALYs rates were observed in the states of Goiás (Central-West Brazil, in the Extra-Amazon region) and Mato Grosso (Central-West Brazil, within the Legal Amazon region). Both these regions received the highest proportion of cases from the North region of the country [16]. In non-endemic areas, the constant presence of infected individuals, along with the persistence of the vector, represents a continuous risk for the reintroduction of the natural transmission [50]. As observed in other states of the Legal Amazon region, Maranhão (which politically belongs to the Northeast region) has been undergoing a process of expansion of its agricultural frontiers since the 1970s and 1980s. This expansion is occurring particularly in the west and north frontiers of the state, in the pre-Amazonian region in the border with the state of Pará. These conditions helped exacerbate malaria transmission in 1986 and 1987 [52].

In the Extra-Amazon region, the state of Piauí (Northeast Brazil) presented the highest incidence rate of malaria in both 1990 and 2017. With the exception of Maranhão, which has part of its territory in the Legal Amazon, the sates in the Northeast of Brazil are considered non-endemic and generally report only sporadic imported cases of malaria. Piauí state records an average of 40 cases of malaria per year, half of which are probably autochthonous. These autochthonous cases are possibly from regions bordering the state of Maranhão [53].

Some authors have pointed out that the spread of malaria to areas outside the Amazon region in Brazil, and particularly to urbanized and industrialized states, is of major concern since these highly populated areas present favorable conditions for the spread of parasites and vectors [13, 54, 55].

In American countries, *P. vivax* is the predominant parasite species and accounted for 74.1% of the cases of malaria in 2017. *P. falciparum* is generally considered the most important etiological agent of malaria in terms of mortality, while *P. vivax* is responsible for the majority of the infections, causing the disease in large areas of the world, including Brazil. An increasing number of reports has argued that the number of deaths due to infections by *P. vivax* is underestimated [7, 56, 57]. It is important to consider the parasite species when estimating disease burden because each species lead to different clinical manifestations of the disease [43, 58]. In this sense, indicators of YLLs due to incapacity and premature death may greatly vary depending on the region evaluated and the predominant parasite species.

Three species of the genus *Plasmodium* were responsible for the 11,327,462 cases of malaria in Brazil between 1990 and 2017. *P. vivax* accounted for 73.5% of the reports, followed by *P. falciparum* (25.5%). One percent of the cases were caused by other species. Noteworthy, *P. vivax* has been increasingly associated with severe malaria, leading to complications that include respiratory distress, shock, and anemia [59–62]. In Brazil, the cases of malaria are seldom caused by *P. malariae*, and those are usually restricted to specific Amazon regions and its surroundings [58, 63]. Therefore, the burden of the disease in the country is a result of the infections by *P. vivax* and *P. falciparum*. What should be emphasized is that each of these two species generates different burdens for malaria [58, 64, 65]. Additionally, factors such as the immunological and genetic characteristics of the exposed population, the climate and environmental conditions, the presence of vector control policies, and the use of antimalarial drugs can influence the burden of the disease [43, 58, 65].

The main goals of the National Malaria Control Program (*Programa Nacional de Controle da Malária*—PNCM) [66] of the Brazilian Ministry of Health are to reduce the case fatality rate and severity of cases, reduce the incidence of the disease, eliminate transmission in urban areas, and maintain the disease controlled in areas where the transmission has already been interrupted. The current scenario in the Amazon region is promising but still requires new approaches to eliminate transmission in the municipalities where it persists. The treatment for malaria adopted by the Brazilian Public Health System (*Sistema Único de Saúde*—SUS) is based on the use of active principles such as chloroquine and primaquine, among others [67].

Even though substantial progress has been made in reducing the burden of malaria in Brazil, planning actions for controlling the disease remains a priority, especially in the Legal Amazon. The risk of death due to malaria results from a combination of environmental, demographic, and others factors, which may result in highly localized risk patterns.

Although the GBD 2017 generated important estimates of the global burden of diseases, the study presents critical limitations regarding the coverage and quality of the Brazilian databases used. Another important limitation resides in the fact that their estimates do not consider the malaria burden caused by *P. falciparum*, *P. vivax*, and *P. malariae* separately. This may be taken into account in future studies, as the three parasite species cause different clinical manifestations, present distinct geographic distributions, and, therefore, should have their specific disease burden estimates [58, 68]. *P. vivax*, for example, can cause multiple relapses, recrudescence, or reinfection following the elimination of infection from the blood due to its stage of hypnozoites in the liver [64, 65, 69, 70]. Thus, it leads to a burden that is different from that caused by the other two parasite species.

Despite these limitations, based on the GBD 2017 estimates, our study showed a declining trend in malaria burden in Brazil during the 27-year study period. However, the disease persists as an important cause of loss of years of healthy life due to premature mortality and disability in the country. Understanding the geographic and temporal distribution of the risk of death and disability is essential for the planning, implementation, and refinement of control strategies aiming to eliminate the diseases.

## Conclusions

The metrics estimated by the GBD 2017 allowed for a better understanding of the burden of malaria in the country and its federated units. Data showed that malaria has affected individuals of both sexes and in 2017 generated similar DALYs rates for different age groups. The highest DALYs rates are concentrated in the North region, likely due to the ecological, social, and migratory conditions that favor the occurrence and circulation of the parasites and the vectors. The persistence of malaria in the Amazon region urges for the constant evaluation of prevention and control measures. Despite the significant decrease in DALY of malaria in the country, it is necessary to maintain control and surveillance measures of the main transmission areas, focusing on the states and municipalities where the disease persists. Resource allocation for further research in the areas and populations most affected by the disease should also be encouraged. In addition, it is necessary to ensure adequate coverage, access, and quality of health services (diagnosis, treatment, and follow-up of cases) to the population in order to prevent the occurrence of severe forms of malaria.

## Data Availability

The estimates of GBD 2017 are available on the GBD study platform at http://vizhub.healthdata.org/gbd-compare and http://ghdx.healthdata.org/gbd-results-tool. The number of disease cases of malaria were obtained from (i) the SUS Epidemiological Report (*Informe Epidemiológico do SUS*) available at http://scielo.iec.gov.br/scielo.php?script=sci_arttext&pid=S0104-16731997000100004, from (ii) the National Health Foundation (*Fundação Nacional de Saúde - FUNASA*) available at http://www.funasa.gov.br/epi/malaria/malaria0.htm, and from (iii) the Epidemiological Surveillance Information System—Case Report (*Sistema de Informação de Vigilância Epidemiológica*—*Notificação de casos*—SIVEP/Malária) available at http://200.214.130.44/sivep_malaria/.

## Abbreviations

GBD: Global Burden of Diseases
YLLs: Years of life lost (due to premature death)
YLDs: Years lived with disability
DALYs: Disability-adjusted life years
UI: Uncertainty intervals
WHO: World Health Organization
CI: Confidence interval
IHME: Institute for Health Metrics and Evaluation
USA: United States of America
ICD: International Classification of Diseases
SIM: *Sistema de Informações sobre Mortalidade*
SINAN: *Sistema de Informação de Agravos de Notificação*
SIVEP: *Sistema de Informação de Vigilância Epidemiológica*
SIH: *Sistema de Informações Hospitalares*
SIA: *Sistema de Informações Ambulatoriais*
SUS: *Sistema Único de Saúde*
CODEm: Cause of Death Ensemble model
NTDs: Neglected tropical diseases
ARC: Annual rates of change
FUNASA: *Fundação Nacional de Saúde*
CAAE: *Certificado de Apresentação para Apreciação Ética*
PNCM: *Programa Nacional de Controle da Malária*
PNPD: *Programa Nacional de Pós-Doutorado*
Capes: *Coordenação de Aperfeiçoamento de Pessoal de Nível Superior*
CNPq: *Conselho Nacional de Desenvolvimento Científico e Tecnológico*
FAPERJ: *Fundação Carlos Chagas de Amparo à Pesquisa do Estado do Rio de Janeiro*
CNE: *Cientistas do Nosso Estado*
PPM: *Programa Pesquisador Mineiro*
